# Fat Embolism Syndrome in Duchenne Muscular Dystrophy Patients: Early Recognition and Aggressive Therapy

**DOI:** 10.1155/2018/3686470

**Published:** 2018-06-04

**Authors:** Lee D. Murphy, Mouhammad Yabrodi, Riad Lutfi

**Affiliations:** ^1^Division of Pediatric Critical Care Medicine, Department of Pediatrics, Indiana University School of Medicine, Indianapolis, IN, USA; ^2^Division of Pediatric Cardiology, Department of Pediatrics, Indiana University School of Medicine, Indianapolis, IN, USA

## Abstract

We describe two pediatric patients with Duchenne muscular dystrophy that presented with acute neurologic deterioration and hypoxic respiratory failure requiring mechanical ventilation. These cases fulfill the clinical criteria for Fat Embolism Syndrome. Early recognition and aggressive supportive therapy with mechanical ventilation, right ventricular afterload reduction, and blood transfusion led to survival without any residual effects from the event. Fat Embolism Syndrome needs to be considered early in the course of patients with Duchenne muscular dystrophy who present with respiratory and neurological symptoms.

## 1. Introduction

Duchenne muscular dystrophy (DMD) is the most common inherited pediatric muscle disorder affecting approximately 1 in 3600 live male births [1]. Patients with DMD have a significant reduction in bone density due to chronic use of steroids, which results in increased incidence of long bone fractures and the potential development of Fat Embolism Syndrome (FES) [2]. When children with DMD present with altered mental status and respiratory compromise, the physician needs to consider FES in the differential diagnosis. We report two cases of pediatric patients with DMD that presented to the emergency department with altered mental status and respiratory compromise that were diagnosed with FES. Due to early recognition of the disease and aggressive supportive care with mechanical ventilation, right ventricular afterload reduction, and blood transfusion, both patients survived without any significant sequelae.

## 2. Case Report

### 2.1. Case 1

A 14-year-old male with DMD has been on daily oral steroid since 9 years of age. He weighed 53 kg (47^th^ percentile) and was 147 cm tall (less than 3^rd^ percentile). He became nonambulatory at 12 years of age. Forced vital capacity (FVC) was 2.37 L or 80% predicted. A polysomnogram was completed and revealed moderate obstructive sleep apnea. His echocardiogram (ECHO) was normal with cardiac magnetic resonance imaging (MRI) demonstrating normal left ventricular ejection fraction (LVEF). He participated in many school activities including wheelchair soccer.

**Figure 1:**
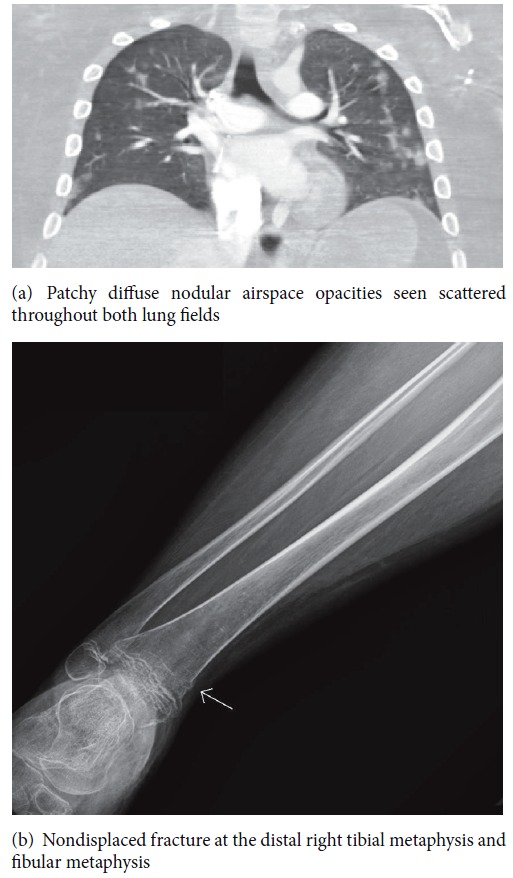


He presented to the emergency department with confusion, tachycardia, tachypnea, and fever up to 39.2 degrees Celsius a few hours following soccer practice. During practice, he had been transferred from his wheelchair and his right leg had brushed the ground causing him to have to be placed on the ground until more help was obtained to put him back in his wheelchair. Brain computed tomography (CT) was negative for any acute intracranial process contributing to his current state. Chest computed tomography (CT) scan was negative for pulmonary embolism but showed patchy diffuse nodular airspace opacities seen scattered throughout both lung fields (Figure 1(a)). He had progressive acute hypoxic respiratory failure requiring mechanical ventilation. Shortly after intubation, he suffered cardiac arrest requiring 3 minutes of cardiopulmonary resuscitation (CPR) before return of spontaneous circulation. ECHOs were consistent with increased pulmonary vascular resistance including moderate right ventricle dysfunction and elevated pulmonary arterial pressure. Duplex ultrasound evaluation of lower extremities was without evidence of deep venous thrombosis bilaterally. X-rays of lower extremities revealed cortical step off at the proximal left femoral neck and nondisplaced fracture at the distal right tibial metaphysis and fibular metaphysis (Figure 1(b)). Ophthalmologic exam revealed Purtscher-like retinopathy. The patient was treated with 48 hours of antibiotics that were discontinued with negative blood, urine, and mini bronchoalveolar lavage (BAL) culture. The patient was treated with mechanical ventilation and right ventricular afterload reduction with milrinone and inhaled nitric oxide for pulmonary hypertension. These therapies were able to be weaned off, and the patient was able to be extubated on day five of admission. He was later transferred to the pediatric floor and later discharged home without any residual effects from the entire event.

### 2.2. Case 2

An 11-year-old male with DMD has been on daily oral steroid since 9 years of age. He weighed 30 kg (5.6^th^ percentile) and he was 120 cm tall (less than 3^rd^ percentile). He is able to ambulate without assistance. FVC was 1.5 L or 96% predicted. His ECHO was normal with cardiac MRI demonstrating normal LVEF.

**Figure 2:**
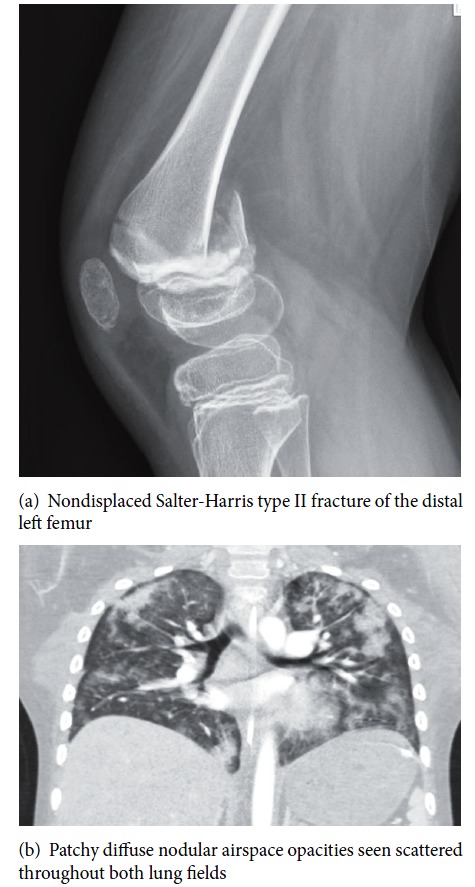


He sustained a nondisplaced Salter-Harris type II fracture of the distal left femur (Figure 2(a)) from a fall that required surgical fixation and was able to be discharged home on the day of operation. He presented to the emergency department two days after his operation with seizure, tachycardia, tachypnea, and fever up to 38.6 degrees Celsius. He had progressive acute hypoxic respiratory failure requiring mechanical ventilation. Shortly after intubation, he developed pulmonary hemorrhage and anemia remedied with fresh frozen plasma, ventilator adjustments, and blood transfusion. A fast spin MRI of his head was negative. CT scan of his chest demonstrated bilateral patchy infiltrates; no pulmonary embolism was identified (Figure 2(b)). ECHO revealed mildly elevated tricuspid regurgitation velocities representing increased right ventricular and pulmonary artery pressures. He was placed on broad spectrum antibiotics for 72 hours but blood, urine, and mini BAL remained negative. Mechanical ventilation was able to be weaned and the patient was extubated on day five of admission. He was later transferred to the pediatric floor and later discharged home without any residual effects from the entire event.

## 3. Discussion

The diagnosis of FES is made up of a group of nondescript symptoms that could represent a multitude of diagnoses [3, 4]. Many cases go unnoticed and therefore the true incidence of FES is unknown [5]. The pathophysiology is thought to be a result of fat droplets released from bone marrow, most commonly after traumatic fractures or orthopedic procedures [4]. Small fat droplets are released into the venous circulation. These droplets can either become lodged in the pulmonary circulation or pass into the systemic circulation and lodge in the cerebral vasculature causing microinfarcts [6]. In DMD patients, FES is increasingly becoming a well-recognized complication as patients with this condition are prone to falls and minor trauma, leading to fractures due to the use of chronic corticosteroids and prolonged immobility [1].

Clinical presentation can range from asymptomatic to cardiac arrest from right ventricular heart failure. Typically, the onset of symptoms happens within the first 12–24 hours after trauma, although some cases may occur as late as 36–72 hours [5, 6]. There are no standardized, prospectively validated diagnostic criteria for FES. The diagnosis is made by recognizing the characteristic clinical syndrome in the context of supportive imaging and a predisposing insult. Given the absence of a gold-standard diagnostic test, a number of authors have proposed clinical diagnostic criteria. The most frequently cited, despite low sensitivity and specificity, are Gurd's criteria (Table 1). Gurd's criteria have been adapted over the years to include the following variations: 1 major with 4 minor or 2 major and 2 minor are the most commonly used definition to diagnose FES [7]. Major criteria include respiratory failure, neurologic changes, and a petechial rash [6, 7].

The lungs are usually the most affected organs with rapidly progressing tachypnea and hypoxemia as the primary clinical symptoms [3, 6]. Chest X-ray shows a diffuse bilateral infiltrate that is hard to differentiate from pediatric acute respiratory distress syndrome. Chest computed tomography (CT) does not add much to a regular chest x-ray in terms of diagnosis; however, it is usually done to rule out pulmonary embolism. Alveolar hemorrhage can be also seen in FES [8].

The brain is the second most affected organ. Neurological symptoms are extremely variable between irritability, anxiety, agitation, confusion, delirium, convulsions and coma, and hypertonia. Cerebral CT scan is usually negative. MRI with DWI is a more specific diagnostic modality [6, 9].

Skin petechiae represent the third most important sign for clinical diagnosis. These tiny lesions (1-2 mm) are, in fact, small hemorrhages caused by the rupture of skin capillaries. The time elapsed from trauma to petechial onset varies with their location but the typical pattern is found in the axillary and high presternal region, in lateral surfaces of the neck, and in eye conjunctiva [5].

In addition to lungs, brain, skin, and conjunctivas, there are minor criteria that include tachycardia, fever, retinal changes, anuria or oliguria, sudden hematocrit and/or platelets drop, and positive fat on sputum [3]. Some patients will develop acute pulmonary hypertension and subsequent acute right heart failure and cardiovascular collapse. Using right ventricular afterload reduction with milrinone and/or inhaled nitric oxide may be beneficial in this setting [10, 11].

A recent systematic review specifically looking at the few published case reports about Fat Embolism Syndrome in children with Duchenne muscular dystrophy found that 7 out of the 16 (44%) children died [12]. In our paper, we report two cases of FES with favorable outcomes. Early recognition and aggressive supportive treatment are key elements for good outcomes in this fragile population. Anticipating potential complications such as anemia, thrombocytopenia, right ventricular failure, or acute kidney injury is another key of the management.

## 4. Conclusion

Our report highlights the need of high index of suspicion for FES in the DMD population and the need for aggressive supportive therapy when these children present with acute altered mental status and respiratory distress.

## Figures and Tables

**Table 1:** Gurd's criteria to diagnose fat embolism syndrome.

	**CASE 1**	**CASE 2**
**MAJOR CRITERIA**		
Petechial Rash	Yes	No
Respiratory Insufficiency	Yes	Yes
Cerebral Involvement	Yes	Yes
**MINOR CRITERIA**		
Tachycardia	Yes	Yes
Pyrexia	Yes	Yes
Retinal changes: fat or petechiae	Yes	No
Jaundice	No	No
Renal: anuria/oliguria or lipiduria	No	No
Sudden fall in hemoglobin concentration	Yes	Yes
Sudden Thrombocytopenia	No	No
High erythrocyte sedimentation Rate	Yes	Yes
Fat macroglobulinemia	No	N/A
